# A mechanically-induced colon cancer cell population shows increased metastatic potential

**DOI:** 10.1186/1476-4598-13-131

**Published:** 2014-05-29

**Authors:** Xin Tang, Theresa B Kuhlenschmidt, Qian Li, Shahjahan Ali, Stephane Lezmi, Hong Chen, Melissa Pires-Alves, William W Laegreid, Taher A Saif, Mark S Kuhlenschmidt

**Affiliations:** 1 Department of Mechanical Science and Engineering, College of Engineering, University of Illinois at Urbana-Champaign, 206 W. Green St, Urbana 61802, Illinois, USA; 2 Department of Pathobiology, College of Veterinary Medicine, University of Illinois at Urbana-Champaign, 2001 S. Lincoln Ave, Urbana 61802, Illinois, USA; 3 Department of Food Science and Human Nutrition and Division of Nutritional Sciences, University of Illinois at Urbana-Champaign, 905 S. Goodwin Ave, Urbana 61802, Illinois, USA; 4 4700 King Abdullah University of Science and Technology, Thuwal 23955-6900, Kingdom of Saudi Arabia

**Keywords:** *In vitro* cancer microenvironment, Metastasis, Mechanotransduction, Cancer biomarkers, Invasiveness, Polyacrylamide hydrogel

## Abstract

**Background:**

Metastasis accounts for the majority of deaths from cancer. Although tumor microenvironment has been shown to have a significant impact on the initiation and/or promotion of metastasis, the mechanism remains elusive. We previously reported that HCT-8 colon cancer cells underwent a phenotypic transition from an adhesive epithelial type (E-cell) to a rounded dissociated type (R-cell) via soft substrate culture, which resembled the initiation of metastasis. The objective of current study was to investigate the molecular and metabolic mechanisms of the E-R transition.

**Methods:**

Global gene expressions of HCT-8 E and R cells were measured by RNA Sequencing (RNA-seq); and the results were further confirmed by real-time PCR. Reactive oxygen species (ROS), anoikis resistance, enzyme activity of aldehyde dehydrogenase 3 family, member A1 (ALDH3A1), and *in vitro* invasion assay were tested on both E and R cells. The deformability of HCT-8 E and R cells was measured by atomic force microscopy (AFM). To study the *in vivo* invasiveness of two cell types, athymic nude mice were intra-splenically injected with HCT-8 E or R cells and sacrificed after 9 weeks. Incidences of tumor development and metastasis were histologically evaluated and analyzed with Fisher’s exact test.

**Results:**

Besides HCT-8, E-R transition on soft substrates was also seen in three other cancer cell lines (HCT116, SW480 colon and DU145 prostate cancer). The expression of some genes, such as ALDH3A1, TNS4, CLDN2, and AKR1B10, which are known to play important roles in cancer cell migration, invasion, proliferation and apoptosis, were increased in HCT-8 R cells. R cells also showed higher ALDH3A1 enzyme activity, higher ROS, higher anoikis resistance, and higher softness than E cells. More importantly, *in vitro* assay and *in vivo* animal models revealed that HCT-8 R cells were more invasive than E cells.

**Conclusions:**

Our comprehensive comparison of HCT-8 E and R cells revealed differences of molecular, phenotypical, and mechanical signatures between the two cell types. To our knowledge, this is the first study that explores the molecular mechanism of E-R transition, which may greatly increase our understanding of the mechanisms of cancer mechanical microenvironment and initiation of cancer metastasis.

## Background

During metastasis, cancer cells escape from the parent tumor, enter the circulatory system, invade host tissues, and form secondary tumors [1-3]. Deciphering the mechanisms initiating metastasis remains elusive due to the difficulty of studying the early stages *in vivo*[3-5]. Cancer cells within the same tumor show a large degree of heterogeneity [4,6] and thus, it is possible that a small fraction of the parent tumor cells are more plastic or stem-like than most others. With appropriate mechanical cues, such as a change in substrate stiffness within the emergent tumor microenvironment, these cells could differentiate into metastatic variants [7,8]. Alternatively, a small population of preexisting metastatic cell variants may be selected for expansion under the influence of appropriate tumor microenvironment [9].

Towards this end, various cancer cell lines, for example those derived from human colon cancer tumors, have been used for *in vitro* studies. Many of these colon cancer cell lines with low metastatic potential (e.g., HCT-8, HCT-116, HT29) are epithelial in phenotype (E cell). When cultured on conventional plastic substrates, they adhere and spread, proliferate, and form E-cadherin-mediated junctions resulting in monolayers covering the entire dish with occasional mounds consisting of 2–3 layers of cells. On top of these mounds or at their vicinity, a variant of the cancer cells is detected [10-14]. These variant cells are spherical in shape, and rare in number (1 rounded-shaped cell per 2 × 10^5^ epithelial-shaped cells). They are called R cells due to their rounded morphology [10,12,13]. Remarkably, the proportion of these R cell variants can be increased by a few orders of magnitude by culturing E cells on appropriately soft substrates. Under these culture conditions 70-90% of the original E cell layers transit to R cells after 17–20 days in culture.

Increasing evidence suggests the mechanical microenvironment plays a role in cancer metastasis [15-20]. For example, a stiffer microenvironment, induced by increased collagen crosslinking in breast cancer tumors *in vivo*, promotes initiation of metastasis [21,22]. An appropriately soft fibrin gel microenvironment produces a metastatic variant of murine B16-F1 melanoma cells that are highly tumorigenic in animal models [23]. It is known that cells generate forces that depend on the stiffness of the microenvironment [20,24,25]. Thus tumor mechanical microenvironments may influence metastatic transition through local force cues that generate or select a subset of metastatic cells. It is then conceivable the multicellular mounds of HCT-8 cells originally observed in conventional cell culture, might have provided a soft microenvironmental niche that facilitated the production of R cells [10-14]. This hypothesis is substantially strengthened by our observation of mass scale production of R cells from E cells on soft substrates [26,27]. We characterized HCT-8 R and E cells by comparing (a) their gene expression profile using RNA-seq, PCR analyses, and selected protein expression using direct enzyme and immunocytochemical analyses, and (b) their *in vitro* invasiveness using cell invasion assays, and *in vivo* metastatic activity in mice using a splenic implantation model. The results imply that R cells are significantly more metastatic than E cells, and the E-R transition induced by growth on soft substrates may offer a new paradigm for simulating the early events of metastasis accelerated by mechanical cues.

## Results

### E-to-R transition in other cell lines cultured on soft substrates

**Figure 1:**
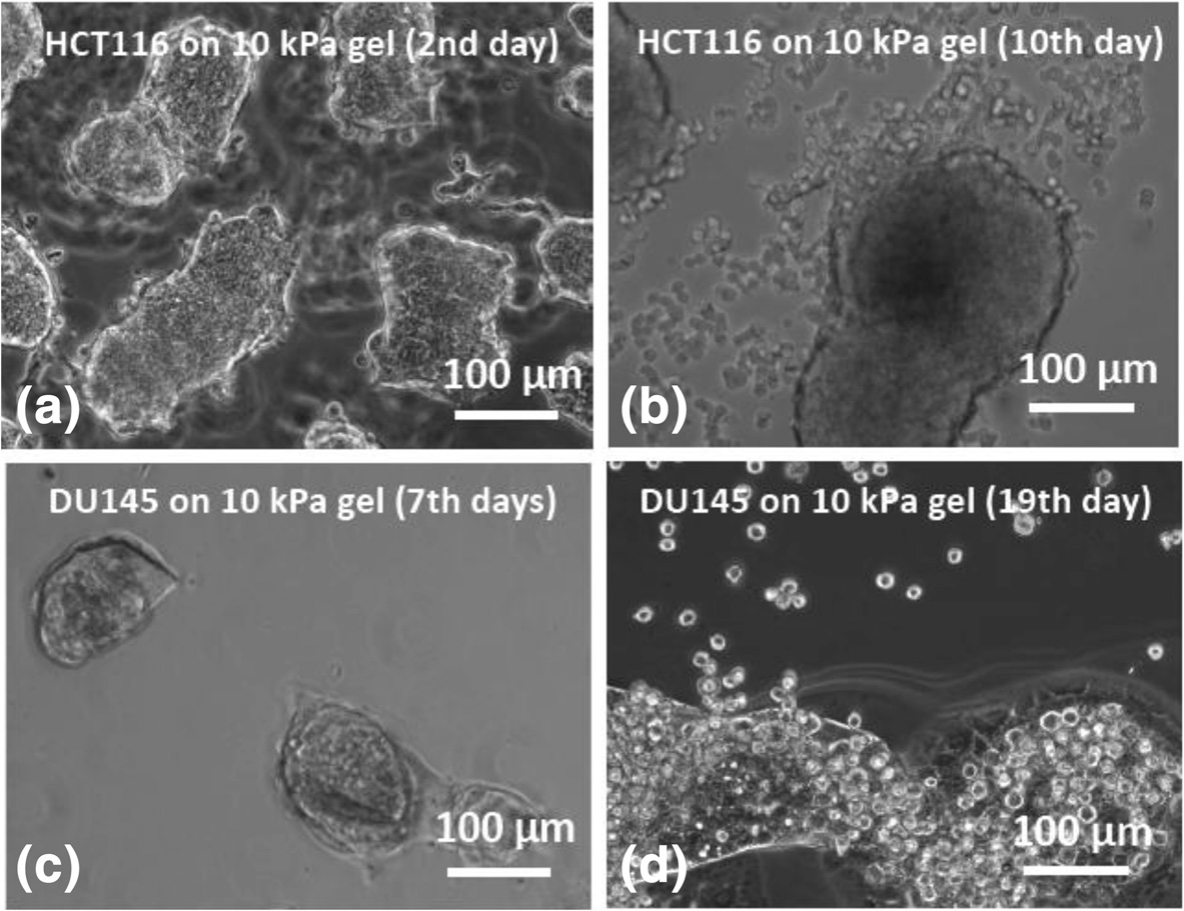
**Multiple cancer cells lines show E-R transition on appropriate soft substrates. (a)** HCT-116 cells cultured on 10 kPa PA gel substrates (coated with fibronectin) form cell colonies in 2–5 culture days. **(b)** HCT-116 cells begin to dissociate from colonies on the 10th day. On PS substrate or other stiffness PA gels under same culture condition, they do not show dissociation. **(c)** DU-145 cells cultured on 10 kPa PA gel substrates (coated with fibronectin) form cell colonies in 3–7 culture days. **(d)** DU-145 cells begin to dissociate from colonies on the 19th day. On PS substrate or other stiffness PA gels under same culture condition, they do not show dissociation. Scale bar: 100 μm.

To explore whether E-R transition is peculiar only to HCT-8 cells, we observed an E-R transition in three other cancer cell lines (HCT116, SW480 colon and DU145 prostate cancer cells) cultured on substrates with various softness. We found colon cancer cell lines, SW480 and HCT116, show E-R transition on 1.0 and 10 kPa gels, respectively, after 10 days of culture, whereas the prostate cancer cell line, DU145, exhibits E-R transition on 10 kPa gel after 19 days (Figure 1). The time points, e.g. 7th or 19th day, are precisely the earliest dates when the first abrupt phenotype change, i.e. cell rounding and dissociation from some (not all) parent cell islands, was observed after the cells were exposed to soft microenvironment. Following initial observation of cell dissociation in any cell island, the majority of all cell islands showed the E-R phenotype within an additional 1–2 days. On hard polystyrene substrates, none of these cells show E-R transition.

To address the potential bias from the difference in surface chemistry between polystyrene and soft gels, polystyrene substrates were incubated with the identical ECM proteins that were coated on soft gels, i.e. Fibronectin, collagen, and laminin, respectively, and then used for cell culture. No E-R transition was observed on these ECM-incubated polystyrene, or the non-ECM-incubated polystyrene substrates. Previously, we reported that the soft gels with appropriate stiffness consistently gave rise to the E-R transition, irrespective of their ECM functionalization [26,27]. Therefore, we reasoned that, while ECM may possibly play a role in regulating some aspects of the E-R transition, the ECM cue was secondary to the substrate stiffness cue in initiation of this process. The results presented here were obtained using the non-ECM-incubated polystyrene.

### RNA-Seq analysis reveals overexpression of metastasis-associated genes in R cells

To further characterize HCT-8 R cells, we performed comparative differential gene expression analyses following E-R transition using whole transcriptome shotgun sequencing (RNA-Seq). Four cell populations, PS3, PS17, Gel3, and R; under selected temporal-spatial culture conditions were compared. The PS3 and PS17 cell populations are HCT-8 cells cultured on polystyrene (PS) substrate for 3 and 17 days, respectively, without any exposure to soft polyacrylamide (PA) gels. Cells under both conditions display the E cell phenotype and do not show an E-R transition. The Gel3 cell population is HCT-8 cells cultured on 20 kPa PA gels for 3 days, but still remained in the E cell phenotype. The R cell population is derived from HCT-8 R cells harvested from 20 kPa PA gels on 17^th^ day, followed by expansion on PS substrate. In an effort to discover differential gene expression patterns in R cells due specifically to the E-R transition, we compared gene expression profiles of R cells with those of Gel 3, PS3, and PS17 cell populations (cell populations not exhibiting an E-R transition). To be scored as a differentially expressed gene, the gene had to satisfy two criteria; 1) observed as differentially expressed in R cells in all three comparisons (R *vs* Gel3, R *vs* PS3, and R *vs* PS17), and 2) could not show differential expression when PS17 and PS3 cell populations were compared. In this manner, we focused on the genes that were more likely differentially expressed due to the E-R transition rather than extended culture time on PS or initial exposure to PA.

**Figure 2:**
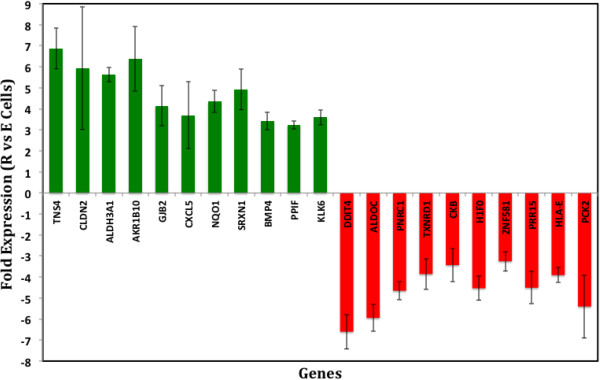
**Summary of differentially expressed genes in HCT-8R cells as compared to E cells.** RNA-Seq analyses were performed as described in Methods. The data are expressed as fold expression. The bar graph represents the average fold expression changes of R cells compared to E cells cultured under different conditions (PS3, Gel3, and PS17) that do not allow an E to R transition. The graphical and tabulated data summarize the up-regulated (green) as compared to down-regulated (red) genes. The composite results suggest that, most of the differentially up-regulated genes in R cells are associated with the functionalities of cell proliferation, motility, metabolism, invasive phenotype, colorectal adenocarcinoma and tumor metastasis. The differentially down-regulated genes in R cells are associated with tumor suppression and inhibition of apoptosis.

We found that 11 genes were up regulated by 3 fold or greater in R cells with respect to those in Gel 3, PS3, and PS17 cells (Figure 1; positive Y axis; q-value < 0.05). These genes are associated with tumor promotion and invasion, repression of apoptosis, cell migration and proliferation, and generation of radical species (Figure 2, Table 1). Noticeably, the colon cancer stem cell marker, ALDH3A1 [28-30], was markedly up regulated in R cells. Two other genes, TNS4 and CLDN2, which are known as clinical markers for colon cancer staging [31-34], also were upregulated in R cells. Ten other genes also were down regulated by 3 fold or greater in R cells (negative Y axis; q-value < 0.05; Figure 2, Table 1). Their functionalities are associated with promotion of apoptosis, maintenance of tissue homeostasis, anti-proliferation, and tumor suppression (Figure 2). Of all those genes, down-regulation of CKB gene was reported to promote epithelial-to-mesenchymal transition (EMT) in colon cancer [35]. These data suggest, following culture on the appropriately soft mechanical microenvironment, a metastasis-enhancing gene pattern is activated in R cells and this activation is likely associated with the characteristics of *in vivo* EMT [3,36,37]. Thus, the E-R transition is exemplified not only by a metastasis-like phenotype, but also a metastasis-like gene expression profile.

**Table 1:** Summary of RNA-seq results showing differentially expressed genes of R cells compared to E cells

**GENE ID**	**GENE**	**AVG**	**SEM**	**R vs PS3**	**R vs Gel 3**	**R vs PS 17**
ENSG00000131746	TNS4	6.87	0.964	7.94	7.24	5.42
ENSG00000165376	CLDN2	5.94	2.92	6.62	9.63	1.56
ENSG00000108602	ALDH3A1	5.62	0.338	5.11	5.87	5.87
ENSG00000198074	AKR1B10	6.38	1.53	8.67	5.19	5.26
ENSG00000165474	GJB2	4.14	0.952	5.27	2.71	4.44
ENSG00000163735	CXCL5	3.69	1.6	5.79	1.29	3.99
ENSG00000181019	NQO1	4.35	0.524	5.13	4.04	3.87
ENSG00000172070	SRXN1	4.92	0.959	6.09	5.18	3.48
ENSG00000125378	BMP4	3.42	0.419	2.79	4.02	3.44
ENSG00000108179	PPIF	3.23	0.184	2.95	3.45	3.28
ENSG00000167755	KLK6	3.59	0.354	4.12	3.41	3.24
ENSG00000168209	DDIT4	-6.6	0.82	-7.53	-5.37	-6.91
ENSG00000109107	ALDOC	-5.95	0.639	-5.19	-6.91	-5.75
ENSG00000146278	PNRC1	-4.66	0.432	-4.01	-4.83	-5.13
ENSG00000198431	TXNRD1	-3.86	0.728	-3.51	-3.12	-4.95
ENSG00000166165	CKB	-3.44	0.792	-2.25	-3.54	-4.52
ENSG00000189060	H1F0	-4.54	0.566	-5.39	-4.06	-4.16
ENSG00000171425	ZNF581	-3.25	0.467	-3.25	-2.55	-3.95
ENSG00000087074	PRR15	-4.51	0.76	-4.44	-5.65	-3.45
ENSG00000204592	HLA-E	-3.91	0.36	-4.01	-4.36	-3.37
ENSG00000100889	PCK2	-5.41	1.5	-7.52	-5.54	-3.16

The tabulated data compare the fold expression changes [up-regulated (positive values) as compared to down-regulated (negative values) genes] of R cells compared to E cells cultured under each of the different conditions (PS3, Gel3, and PS17) that do not allow an E to R transition.

### RT-qPCR validation of differential gene expression

**Figure 3:**
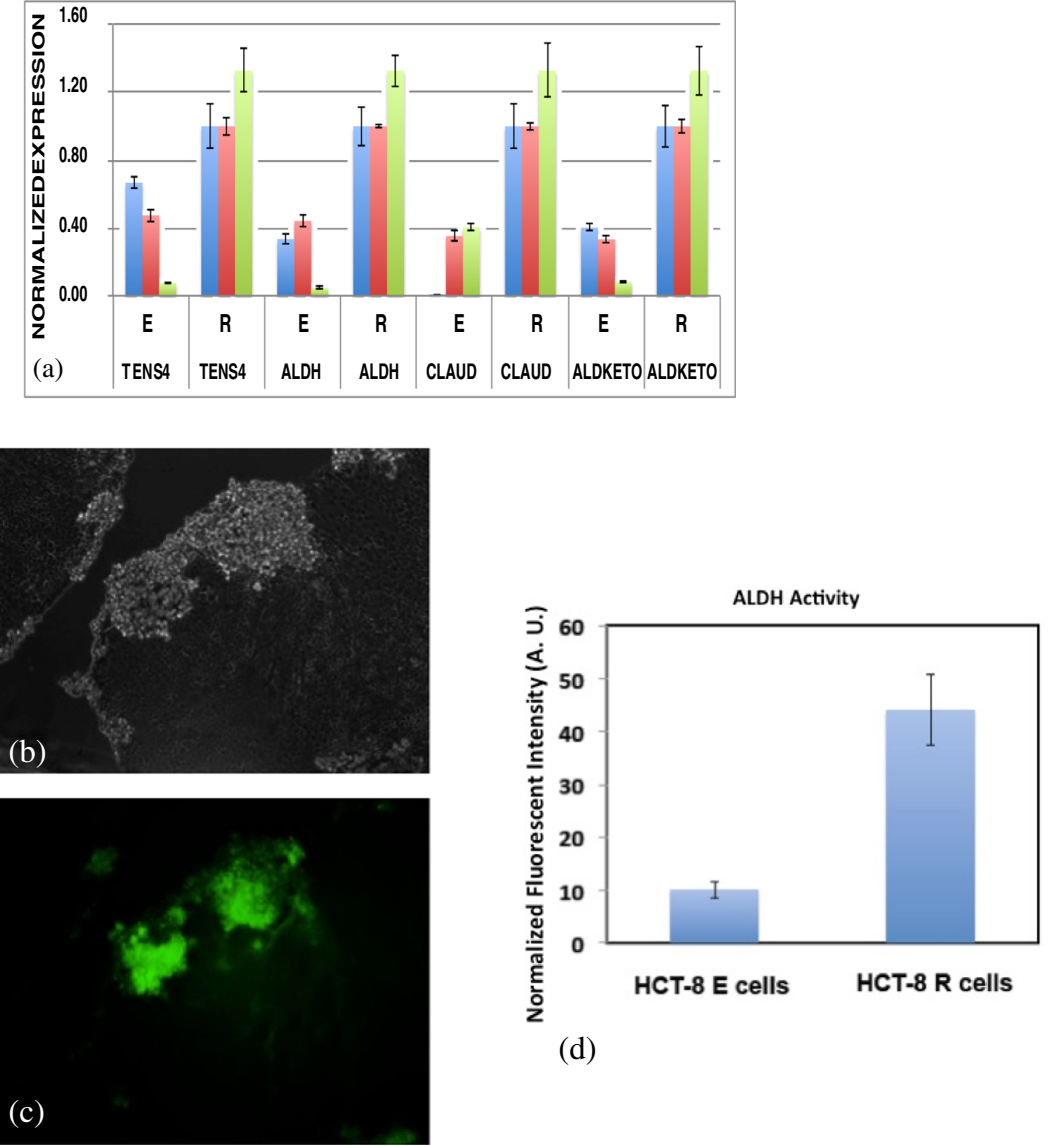
**Verification of selected gene and protein expression.** Verification of selected gene and protein expression was performed using RT-qPCR and direct ALDH enzyme-staining assays as described in Methods. **(a)** RT-qPCR was used to verify overexpression of selected genes identified by RNA-Seq analyses. The blue, pink, and green bars represent 3 separate experiments each consisting of 3 replicate samples for each primer set. One-way ANOVA analyses indicate the mean value increases in gene expression between E and R cells in all experiments for TENS4 (p < 0.03), ALDH3A1 (p < 0.01), CLDN-2 (p < 0.01) and AKR1B10 (P < 0.005) represent statistically significant differences. **(b-c)** fluorometric ALDH enzyme assays were used to directly measure the relative expression of ALDH in R and E cells in cell monolayers undergoing E-R transition on soft substrates. 90% of R cells, as well as some E cells in the cell islands undergoing E-R transition express relatively high amounts of ALDH activity. Scale bar: 100 μm. **(d)** The integrated cellular fluorescence intensity of ALDH for R cells was 4–5 fold higher as compared to E cells.

To confirm the RNA-Seq results, we used RT-qPCR to verify the differential expression of some selected up-regulated genes in R cells. The representative genes used were ALDH3A1, TNS4, CLDN2, and ALDKETO (Figure 2), and are known to play important roles in cancer cell de-differentiation, migration, invasion, proliferation and apoptosis suppression [29-34,38-40]. Our qPCR results are consistent with the over-expression of these genes as observed in the RNA-Seq experiments (Figure 3a).

### Dissociated R cells express an enzymatically active cancer stem cell marker

ALDH3A1 was identified by RNA-Seq and RT-qPCR as over expressed in R cells. ALDH3A1 has been identified as an important enzyme serving as a marker of various types of cancer stem cells [30,41-43]. In addition to confirming its over-expression in R cells, we also used direct enzyme activity staining to test whether R cells or E cells on soft substrates possess increased ALDH3A1 enzyme activity. We found greater than 90% of R cells, as well as a small portion of E cells in the cell islands prior to E-R transition, show high levels of ALDH3A1 (Figure 3b-c). The integrated whole-cell fluorescent intensities of ALDH3A1 for R cells are 4–5 fold higher than E cells (Figure 3d). These results indicate, in addition to its high mRNA expression, ALDH3A1 enzyme activity is enhanced in R cells. These results also suggest that *in vitro* culture on soft substrates may promote the selection or production of cancer stem cells.

### Reactive oxygen species (ROS) production is up regulated in R cells

**Figure 4:**
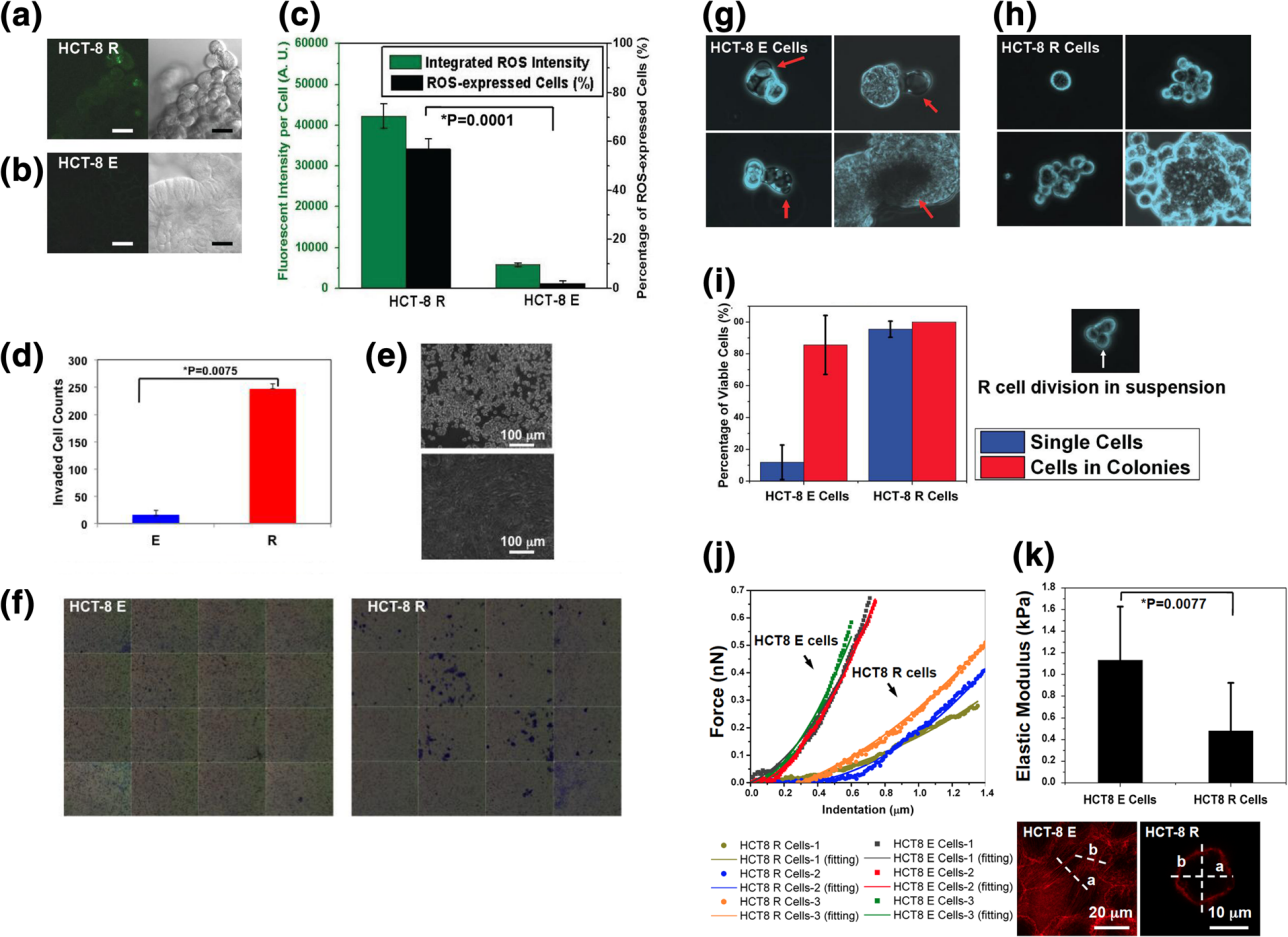
**Biophysical properties of E and R cells. (a-b)** Reactive oxygen species of HCT-8 E and R cells detected by staining with 5-(and-6)-carboxy-2′, 7′-dichlorodihydrofluorescein diacetate. Left: Fluorescent pictures. Right: DIC pictures. Scale bar: 20 μm. **(c)** Comparison of ROS production in E and R cells. **(d)** Comparison of basement membrane invasiveness of E and R cells. R is HCT-8 R cells harvested from 20 kPa gels on after 17 days culture. E is HCT-8 cells cultured on PS for 10 days. **(e)** Phase-contrast photomicrographs of HCT-8 R (top) and E cells (bottom) cultured on PS prior to harvesting and invasion assay. Scale bar: 100 μm. **(f)** Assembled 4x4 picture set of invaded HCT-8 E cells after 48 hr incubation. Assembled 4x4 picture set of invaded HCT-8 R cells after 48 hr incubation (purple foci indicates invaded cells). **(g-h)** Comparison of the anchorage independent growth and viability of HCT-8 E **(g)** and R cells after culture on non-functionalized gels for 8 days. The percentages of viable E and R cells were determined by Trypan-blue staining **(i)**. Red arrows in **(g)** indicate the dead cells. **(j)** Atomic force microscopy measurement of stiffness of HCT-8 cells before and after E-R transition. The square-dot and circle-dot curves represent the force vs. indentation displacement of HCT-8 E and R cells, respectively. The data were fitted using an improved Hertz model to extract the Elastic modulus of cells. **(j)** Comparison of the cell elasticity of E and R cells. **(k)** Comparison of actin organization in E and R cells on PS. Rhodamine phalloidin (520/650, red) was used specifically stain F-actin filaments. The spatial fluorescent intensity distributions of actin expression across E and R cells body are indicated by dashed lines a and b). Scale bar: 10 μm.

The RNA-Seq gene expression profile showed, following E-R transition, R cells express genes associated with cell survival and inhibition of apoptosis. It is known that the activation of cell survival signaling pathways is often accompanied with the release of ROS from cell mitochondria [44-46]. To determine relative ROS expression, we used a stable fluorogenic marker, 5-(and-6)-carboxy-2′, 7′-dichlorodihydrofluorescein diacetate (carboxy-H_2_DCFDA) to stain both HCT-8 E and R cells followed by imaging using high-resolution multi-photon confocal microscopy. The marker distinguishes the oxidatively stressed and non-stressed cell populations through fluorescent-intensity ratio-metrics (Figure 4a-c). Our data show 57.8 ± 5.84% of R cells exhibit high ROS expression (fluorescent intensity = 40000 ± 5000 (A. U.)). Only 2.32 ± 1.07% of E cells express weak ROS (maximum fluorescent intensity = 5000 ± 300 (A. U.)). The integrated whole-cell fluorescent intensity in R cells is 6–8 fold higher than in E cells (p-value = 0.0001; Figure 4c). It is known that expression of ROS can facilitate cell proliferation, mutagenesis, evasion of apoptosis and malignant transformation [47,48].

### R cells display enhanced basement membrane invasion

We compared the invasiveness of E and R cells using an *in vitro* cell invasion assay. HCT-8 R cells were harvested from 20 kPa PA gels on 17^th^ culture day following E-R transition and expanded on PS substrates for another 7 days before the invasion assay (Figure 4d-e). HCT-8 E cells were cultured on PS substrate for 10 days prior to the invasion assay (Figure 4e). The results of these *in vitro* invasion experiments show that R cells are significantly more invasive, an essential metastatic phenotype [3,9,49], compared to E cells: the number of R cell foci penetrating through basement membranes is more than 10 fold greater than observed with E cells (P value = 0.0075; Figure 3d).

### R cells acquire anoikis resistance

A critical step for successful metastasis is the survival of cancer cells in absence of substrate attachment [50]. Normal cells undergo programmed cell death, i.e., anoikis, when kept unattached to a substrate matrix or neighboring cells. Anoikis, a physiologically essential function, prevents detached cells from forming dysplastic colonies at places other than their correct anatomical locations, and ensures tissue homeostasis [50-52]. Metastasizing cancer cells, however, acquire the ability of anoikis resistance and survive even after detaching from the primary tumor; an ability that enhances their dissemination through the circulatory and lymphatic systems [51,53]. To test whether HCT-8 R cells have acquired anoikis resistance, we performed cell suspension growth assays for both R and E cells. PA gels without any ECM coating were used to culture both cell types for 8 days followed by cell viability measurements. Since ECM-free gel surfaces are inert and non-adhesive, cells do not form any specific or non-specific anchorage and are thus kept in suspension. The anchorage-free growth and viability of cells on gels were confirmed by daily imaging.

After 7 days culture in suspension, 95.4 ± 5.07% of single HCT-8 R cells were alive (Figure 4i), and maintained cell proliferation (Figure 4h lower panel) throughout this suspension culture period. In contrast, only 11.8 ± 10.9% of single HCT-8 E cells were alive after the same period of suspension culture (P value = 0.0001, Figure 4i). As E cells form aggregates in suspension, their viability increased 7–8 fold, and 85.6 ± 18.6% of the E-cell aggregates were alive, indicating that cell-cell contact might trigger certain survival pathways to partially evade anoikis *in vitro* (Figure 4g) [54]. For R cells in loosely packed aggregates (Figure 4h), their viability was not significantly different from those in single cell form, and their cell division was not impaired due to suspension culture (Figure 4h, lower panel). Furthermore, the R cell numbers in suspension increased significantly due to sustained proliferation as the suspension culture continued (Figure 4i). These results are consistent with the RNA-Seq data, indicating R cells up-regulate apoptosis inhibition gene expression compared to E cells. The results suggest R cells are potentially capable of surviving and growing *in vivo* as would be expected of a metastasizing cancer cell.

### R cells are mechanically softer

Cancer cells with higher metastatic potential generally display greater deformability [55-59], which allows their easy transit through vasculature during metastasis. Thus, we compared deformability of HCT-8 E and R cells using the contact mode of atomic force microscopy (AFM). HCT-8 E and R cells were both cultured on PS substrates under identical environment conditions (37°C) throughout AFM analysis (Figure 4j, k and Methods section). We found R cells are 2–3 times softer, with Elastic modulus = 0.47861 ± 0.44339 kPa (n = 8), than the E cells with Elastic modulus = 1.13107 ± 0.49646 kPa (n = 12; P value = 0.0077; Figure 4j). The softened R cell elasticity agrees with the previously reported altered actin cytoskeleton architecture in R cells [26]. Specifically, on hard PS substrates E cells show well-organized, straight-ordered actin stress bundles within individual cells. In contrast, HCT-8 R cells show only cortical actin structure with no actin stress bundles, implying a more compliant state (Figure 4k).

### R cells display increased *in vivo* metastatic activity compared to E cells

**Figure 5:**
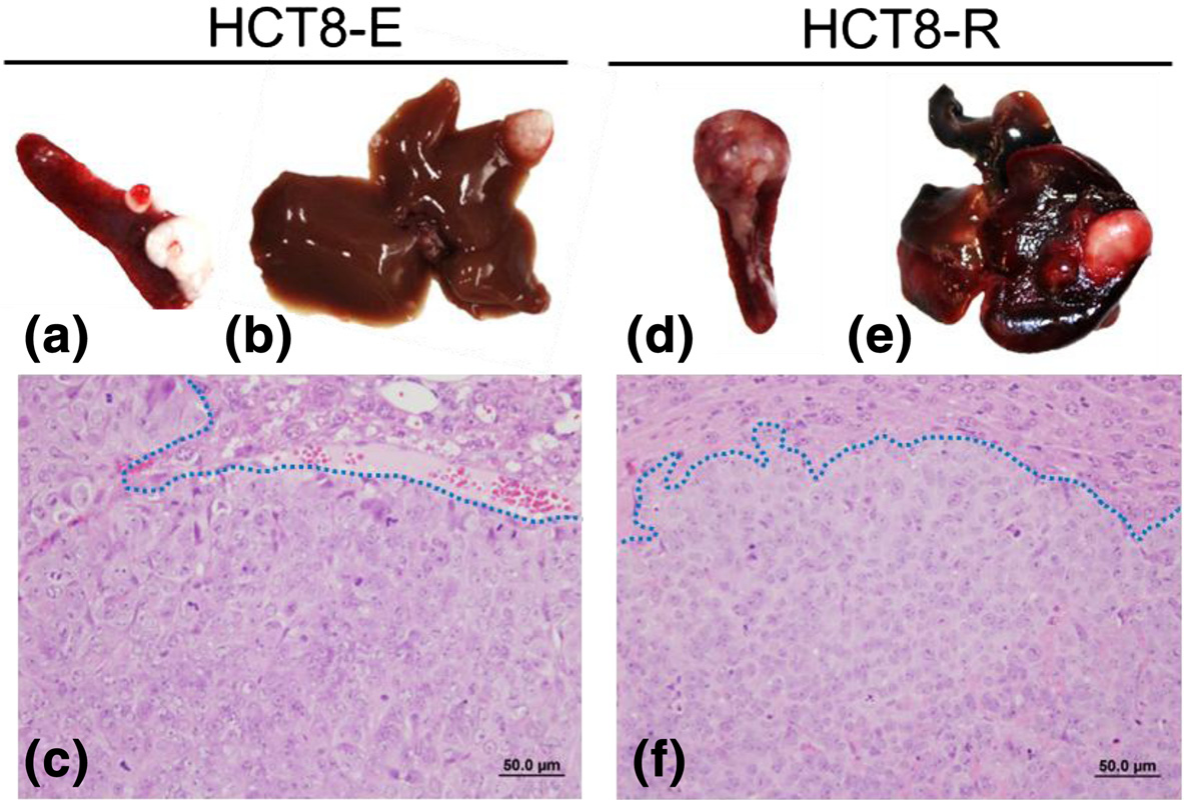
**Macroscopic and microscopic aspects of tumors.** Macroscopic illustration of tumors in the spleen **(a & d)** and liver **(b & e)** of HCT8 cell-injected nude mice. **c & f** illustrate the similar histological growth pattern and cellular morphology of HCT-8E **(c)** and HCT-8R **(f)** cells in the liver (H&E staining, dots show the division of the liver tissue on the top and tumor on the bottom).

In order to evaluate *in vivo* hepatic invasiveness and metastatic potential, HCT-8 E and R cells were surgically injected into the spleen of athymic nude mice. After 9–10 weeks, all animals were euthanized and sacrificed. Spleen, liver, and other tissues with tumor development (Figure 5a, b, d, and e) were fixed in formalin and embedded in paraffin to prepare histological slides (Figure 5c, f). Incidences of tumor development and metastasis between HCT-8 E and HCT-8 R groups were evaluated with Fisher’s exact test. Necropsy and histological evaluations showed 69% of the mice injected with HCT-8 R cells and 71% injected with HCT-8 E cells developed tumor(s), indicating a similar rate of tumor implantations/development (Table 2). On the other hand, 73% of mice injected with HCT-8 R cells had more than one implantation sites and/or metastases, compared to 40% for mice injected with HCT-8 E cells (Table 2). Regarding liver metastases, there was a mean of 5.1 metastases in the liver of HCT8-R-injected mice and 3.4 metastases in HCT8-E-injected mice (Table 2). The mitotic rate (number of mitoses per 400× magnification field), reflecting the growth of neoplastic cells in tumors, was identical in both HCT-8 E and HCT-8 R groups (Table 2). Interestingly, the rate of implantation of tumors in mice injected with E or R cells were similar. Also, there were no morphological differences (growth pattern, stroma collagen production, mitotic index) between R and E cell tumors in our *vivo* model. Thus, the higher rate of tumor implantation with R cells was not due to cell survival in the spleen after injection or to different growth capacity of neoplastic cells, but likely due to higher migration and invasiveness. These results suggested that HCT-8R cells were more inclined to spread and migrate to different organs compared to E cells.

**Table 2:** Comparison of tumor development in nude mice

**Cell types**	**Number of animals**	**Mice (%) developing tumors**	**Mice (%) with more than one implantation site**	**Mean number of metastases in the liver**	**Mean mitotic rate**
**HCT8-E**	**7**	**71 (5/7)**	**40**	**3.4**	**7.6** ± 2.0
**HCT8-R**	**16**	**69 (11/16)**	**73**	**5.1**	**7.3** ± 2.7

HCT8-E and HCT8-R cells were injected into the spleen of nude mice as described in Methods. Spleen tumor implantation and liver metastases numbers were based on the evaluation of H&E stained slides following 9–10 weeks after injection.

## Discussion

In this paper, we have demonstrated that soft substrate culture for 7–17 days can elicit remarkable phenotypic and functional changes in HCT-8 colon cancer cells. Soft substrate culture results in a cell population that exhibits a rounded shape, increased basement membrane invasion, anchorage-independent growth, and differential expression of genes associated with increased tumorigenicity, invasiveness, cell survival, and *in vivo* metastasis. In our *in vivo* metastatic animal model, HCT-8 R cells acquired strikingly efficient tumorigenic capacity (i.e. capacity to produce tumors in multiple organs) after injection. This mechanically-induced transition is not only limited to HCT-8 cells, as we observed the transition in other cancer cells lines as well, although the time to transition and the optimum substrate softness were dissimilar. These results, combined with our molecular data, suggest the R cells can be reasonably considered as a metastatic cell variant. Similar data were obtained by others with a rounded melanoma cell line, previously cultured on 3D soft fibrin gels, that were highly tumorigenic (tumor development with very low numbers of cells injected subcutaneously or intravenously in mice) [23]. Our model clearly demonstrates that colon cancer R cells, produced from E cells through soft substrate culture, displayed a metastatic phenotype that was not linked to cell survival or mitotic rate of neoplastic cells *in vivo*. Our results raise the possibility that cells from different origins may undergo an accelerated metastatic transition dependent on their respective optimum mechanical microenvironmental niche.

Our RNA-seq and protein expression analysis showed that a soft mechanical microenvironment stimulated HCT-8 cells to express a number of metastasis-enhancing genes associated with functions such as apoptosis inhibition, motility, metastatic activity and cancer stem cell traits. Among those, ALDH3A1, CLDN2 and TNS4 (CTEN), were found consistently up-regulated in R cells, are of particular interest.

Aldehyde dehydrogenases (ALDHs) are cytosolic enzymes responsible for oxidizing aliphatic and aromatic aldehydes to carboxylic acids. The expression of the ALDH isoform 3A1 (ALDH3A1) has been used as a cancer stem/progenitor marker from multiple organs, including colon and liver [39,60-64]. High ALDH3A1 expression and activity have also been found to be closely correlated with cell proliferation [65], increased cell metabolism [66], and prevention of apoptosis [30,41]. Interestingly, in our model, R cells showed prolonged survival in suspension, and resistance to apoptosis with high elevation of ROS that likely correspond to acquired important traits for successful metastasis.

CLDN2 is a transmembrane protein that has been shown to result in epithelial permeability, neoplastic transformation, significant increases in cell proliferation and anchorage-independent growth when overexpressed in colorectal cancer cells [32]. CTEN plays an essential role in regulating the integrin-actin cytoskeletal organization and enhancing cancer cell motility and anchorage-independent growth [33,67]. Interestingly, CTEN lacks the N-terminal actin binding domain and its induction has been associated with a reduction in E-cadherin protein expression (but not the amounts of E-cadherin mRNA) [31] as well as displacement of tensin-3 from the cytoplasmic tail of integrin β_1_ causing actin fiber dissociation and increased cell motility, invasiveness, and metastasis [33]. These results parallel our previous observation of HCT-8R cells reducing E-cadherin expression [26,27] and the current observation of the R cells showing a 7-fold increase in CTEN mRNA expression. Taken together, these results are consistent with the hypothesis that a switch toward CTEN rather than tensin-3 expression is associated with increased colon cancer cell metastasis.

Interestingly, in human patients affected with colon carcinoma, these genes (ALDH3A1, CLDN2 and CTEN) also have been associated with poor clinical outcomes. Indeed, ALDH3A1 expression and activity has been found to be closely correlated with resistance to apoptosis induced by cytotoxic effects [65,68] or by some chemotherapeutic agents, such as cyclophosphamide [69,70] and oxazphosphorines [71]. The analysis of mRNA and protein expression using a total of 309 patient samples revealed that CLDN2 expression is significantly increased in colorectal cancer, and correlates with cancer progression and tissue invasion [32,33,67]. It has also been reported that colon cancer tumors with higher CTEN mRNA expression display a poorer histological grade, deeper serosa invasion, and more positive lymph node metastasis or peritoneal dissemination. Multivariate analysis also revealed high CTEN mRNA expression could serve as poor prognostic predictor for colon cancer staging [34].

It is rather surprising to discover that HCT-8 R cells harvested following weeks of culture on soft substrates show *in vitro* irreversibility of their rounded morphology and altered expression of proteins such as E-Cadherin and actin cytoskeleton filaments [26,27]. This interesting phenomenon raises another question: Is it a transition or selection process promoted by soft substrates? Instead of initiating E-R transition *de novo*, it is possible that soft substrates might enhance the selection of a pre-existing R cell population that possesses the rounded morphology and invasive characteristics seen in our results. The results of RNAseq analyses, qPCR, and enzyme activity studies consistently show that R cells express the cancer stem cell marker ALDH3A1. Recently it was reported that an appropriately soft fibrin gel microenvironment selects a metastatic variant of murine B16-F1 melanoma cells that possess the traits of cancer stem cells and are highly tumorigenic in animal models [23]. During culture of HCT-8 E cells in standard tissue culture flasks, we and other independent groups [10-14] also showed that a few rounded isolated cells (R-like cells) appeared only on top of confluent epithelial monolayer (E cells). Although future work is needed to investigate the similarity between pre-existing R cells and R cells produced by E-R transition and dissociation from E-cell islands on soft gels, we cannot rule out the intriguing possibility that soft substrates might enhance the selection and proliferation of a pre-existing stem-like R cell population. Stem-like R cells, instead of epithelial-like E cells, might have preferred proliferation on the mechanically soft microenvironment. This hypothesis; however, appears to somewhat contradict our experimental observations showing that only R cells were present on soft PA gels after 2–3 weeks of culture, while there were nearly no E cells remaining. It seems unlikely that a selection process would result in almost complete elimination of the E cell population. Furthermore, the morphological appearance of R-cell generation from the edge of E-cell islands (26) appears more consistent with a conversion (E-R transition) than a selection process. Nevertheless, it is quite possible that both a selection and a transition processes might co-exist.

The underlying mechanism by which cancer cells sense the microenvironment and decide their metastatic fate remains elusive, but inter- and intracellular forces are likely to play critical roles. The metastatic transition we have demonstrated *in vitro* immediately enables us to explore the physical-molecular mechanisms of the tumor microenvironment that may initiate the metastatic journey of cancer cells by turning on the necessary molecular pathways. Understanding these mechanisms may lead to the engineering of novel therapeutic drugs capable of inhibiting the initiation of metastasis, for example by blocking the downstream molecular pathways up regulated by the changing tumor mechanical microenvironment. Furthermore, new molecular biomarkers may be developed for rapid, earlier detection and monitoring of metastatic activity.

## Conclusions

Our comprehensive, comparative investigation reveals HCT-8 R cells are remarkably more invasive and tumorigenic compared to E cells. The R cells express many of the molecular signatures associated with resistance to hypoxia, apoptosis, as well as genes linked to metastasis and poor clinical outcome in colon cancer patients. This E-R transition, accelerated by *in vitro* mechanical cues, may mimic the early stages of metastasis, which are difficult to study *in vivo*. The study presented here suggests mechanical forces within the tumor microenvironment may play a pivotal role in early metastatic transition, and such mechanical environments can be mimicked in an *in vitro* model. Such *in vitro* models may enable us to identify the early cellular, mechanical, biophysical, molecular and genetic events responsible for initiation of cancer metastasis, assess the relative metastatic potential of cells obtained from patient biospecimens, identify molecular markers for early diagnosis, and to screen putative therapeutics for inhibition of metastasis.

## Methods

### Cell culture

Human colon adenocarcinoma HCT-8 cells (Cat. No. CCL-244; ATCC, Manassas, VA) were cultured as previously described [26,27,72]. Human colon adenocarcinoma SW480 cells (Cat. No. CCL-228; ATCC, Manassas, VA) were cultured in L-15 Medium (Cat. No. 30–2008; ATCC) supplemented with 10% fetal bovine serum and Penicillin Streptomycin. Human colon carcinoma HCT-116 cells (Cat. No. CCL-247; ATCC, Manassas, VA) were cultured in McCoy’s 5a Medium (Cat. No. 30–2007; ATCC) supplemented with 10% fetal bovine serum and 1× antibiotic-antimycotic (Cat. No. 15240–062; Gibco). All cells were cultured at 37°C, with optimal humidity and 5% CO2.

### PA gels preparations and AFM calibration

Polyacrylamide (PA) gels were prepared following the protocols described [26,73-76]. The PA gels are made with varied relative concentrations of acrylamide (Bio-Rad) and N, N’- methylene bis-acrylamide (Bio-Rad Inc.) to obtain different cross-link percentages resulting in different elastic moduli. For 20 kPa PA gels, the mol/v concentrations of acrylamide and N, N’- methylene bis-acrylamide are 8% and 0.13%, respectively. All gels, except the ones used for suspension tests, were covalently coated with 25 μg /mL fibronectin (BD Science Inc.). Atomic force microscopy (Asylum), with silicon-nitride cantilever having a spring constant k = 148.14 pN × nm-1 (Veeco) and equipped with the environmental chamber, was used to characterize the stiffness of PA gels as well as HCT-8 E and R cells. A conical tip approximation for the AFM tip was used to extract the Elastic modulus of cells and gels [26,77].

### Basement membrane invasion assay

HCT-8 E cells were grown and HCT-8 R cells were produced on soft PA gel substrates (20 kPa), and harvested as previously described [26,27]. During harvesting, the R cells from PA gels were flushed from the gel surface using sterile culture media and filtered using a sterile 40 μm mesh filter (BD Falcon #352340) to remove primarily residual E cell clusters. E cells and R cells (following expansion on PS for 7 additional days) were harvested with trypsin EDTA and all cell populations adjusted to the same starting concentration (7 × 106 cells per ml)]. Aliquots (0.3 ml) of the starting R or E cell concentrations were applied to the upper compartment of a CytoSelect™ Cell Invasion chamber (Cat #: CBA 110, Cell Biolabs). Following 48 hours of culture in the assay chambers invasive cells are able to degrade the matrix proteins in the layer, pass through the pores of the polycarbonate membrane, and adhere to the bottom ECM membrane insert. Non-invasive cells were removed from the upper chamber and invasive cells exposed on the bottom of the ECM membrane were stained according to the manufacturer’s instructions and quantified by a light microscope using Metamorph imaging software.

### Whole transcriptome shotgun sequencing (RNA-Seq) and pathway analysis

Whole Transcriptome Sequencing (RNA-Seq) analysis was carried out for RNA derived from cells cultured on soft PA gel and hard polystyrene (PS) substrates. Harvested cells were put in RNA protect reagents (Qiagen Inc., Valencia, CA) to preserve the integrity of RNA. Total RNA samples were isolated from E and R HCT-8 cells using RNeasy Mini Kit (Qiagen Inc., Valencia, CA) according to manufacturer’s recommendation. Briefly, confluent E cells at passage 18 (approximately 3-4 × 10^6^ cells per P60 polystyrene dish) following 3 or 17 days of culture were rinsed with PBS followed by scraping off the cell layer in 750 μl of RNA Protect. Cells were pelleted by centrifugation. Cell pellets were suspended in 350 ul of RLT buffer from the RNeasy kit and were homogenized using the Qiashredder spin column. The homogenized cell lysate effluent from the Qiashredder spin column was then processed as per RNeasy mini kit manufacturer’s directions, which included a gDNA eliminator column and total RNA was obtained in water. Likewise, total RNA from E cells grown on 20 kPA polyacrylamide gels for 3 days was obtained. Total RNA from R cells was harvested following growth on 20 kPA polyacrylamide gels for 15 days followed by filtering through 40 μm mesh (BD Falcon #352340) and expanded by culture on PS for 4 days. Concentrations and initial quality assessment of the RNA were determined by measuring absorbance at 260, 230 and 280 nm. RNA quality of the samples was checked using Nano6000/Pico bioanalyzer chips (Agilent 2100 Bioanalyzer, Santa Clara, CA) at the Keck Center for Comparative and Functional Genomics, University of Illinois. These RNA samples are denoted as PS3 (culture on polystyrene for 3 days), PS17 (culture on polystyrene for 17 days), Gel3 (culture on polyacrylamide for 3 days), and R (cells harvested after 15 days of culture on 20 kPA gels and expanded on PS for 4 days).

Following RNA isolation an additional DNase1 digestion step was performed to ensure that the samples are free from any contaminated with genomic DNA. Libraries are prepared using TruSeq RNA Sample Prep kits (Illumina Inc, San Diego, CA) and sequencing is performed at the King Abdullah University of Science and Technology (KAUST), Saudi Arabia, using an Illumina HiSeq2000 sequencer. Sequence run was then set up for 100, 7, and 100 cycles for Read1, Index Read and Read2, respectively on HiSeq2000 using TruSeq SBS V3.0 kit. Off Line Base (OLB) calling, demultiplexing and FastQ files were then generated using CASAVA 1.8.2 (Illumina Inc. San Diego, CA, USA).

E and R cell RNA-Seq reads are trimmed to remove poor quality sequences in Roy J. Carver Biotechnology Center, UIUC. Sequence files are mapped against the Human Genome (hg19) using TopHat tool such that only uniquely mapped reads are reported and used for further data analysis. Splice junctions are automatically determined by TopHat, with the provided guidance of annotated gene models (GTF file) obtained mainly from Ensembl. In this analysis, all three splice sites, “GT-AG”, “GC-AG” and “AT-AC” will be considered. All splicing junctions supported by at least one high-quality mapped read will be kept. The resulting alignment data from Tophat are then fed to an assembler, Cufflinks, to assemble aligned RNA-Seq reads into parsimonious set of transcripts and to estimate their abundances and test for differential expression and regulation in RNA-Seq samples. Cufflinks is used to address the common issue of read alignment to multiple isoforms of the same gene or multiple transcripts within the same genetic locus in the assembly by performing maximum likelihood estimation based on a numerical optimization algorithm for calculating FPKM. Cufflinks is executed for each sample separately using their alignment file (.BAM file) and hg19 annotation file (.gtf file), which results in a ‘transcripts.gtf’ files for each sample. Cuffcompare tool from Cufflinks package is used to track the cufflinks transcripts (transcripts.gtf) across multiple experiments and compare the assembled transcripts across all samples to the reference annotation file (.gtf file) and to give a combined (.gtf) file. Cuffdiff tool from Cufflinks package will be used to find significant changes in gene expression. This tool is executed using alignment files of two samples being compared and using combined .gtf files generated from previous cuffcompare tool. Transcript abundances are measured in Fragments Per Kilobase of exon per Million fragments mapped (FPKM), which originate from RPKM (Reads per Kilobase per Million). Student’s t-test was then used to find significantly differentially expressed transcripts, with the test statistic (fold expression) derived from the log ratio of FPKM values when comparing two samples. In an attempt to bias the results to screen for gene expression changes primarily resulting from the transition from an E to R cell induced by a change in substrate mechanical stiffness rather than variance in general cell culture conditions, genes were scored as differentially expressed only if they were seen simultaneously in three different comparisons (R vs PS3, R vs Gel3 and R vs PS17). These three comparisons represent comparing cells that have undergone E to R transition (R cells) with E cells that were exposed to either polystyrene (hard) or polyacrylamide gels (soft) substrates for varying culture times but that did not undergo E to R transition. Preliminary pathway analysis was carried out using the online Panther software with focus on the genes with differential expression comparison levels lower than q-value of 0.05 and according to the criteria mentioned above.

### RT-qPCR

First strand c-DNA was synthesized from total RNA using Superscript III catalog # 18080–051 (Invitrogen Life Technologies, Grand Island, NY) according to manufacturer’s direction. Quantitative RT-PCR (RT-qPCR) was performed using SsoFast Eva Green Supermix system (catalog number 172–5200, Bio-Rad Laboratories, Hercules, CA) according to manufacturer’s directions. Samples were heated at 95°C for 15 min then subjected to 40 thermocycles (95°C for 5 seconds, 60°C for 10 seconds, 72°C for 30 seconds, followed by a 60 to 95°C melt curve) of PCR amplification using an MJ Mini Opticon real time PCR detection system (Bio-Rad laboratories, Hercules, CA). Triplicate reactions were performed for each sample. PCR products were analyzed using Ethidium Bromide stained 2% agarose gels (E-gels, Invitrogen, catalog number G5018-02) and a 50 bp ladder standard from Biolabs N0473G.

Primer design was done using program Primer 3 (http://biotools.umassmed.edu/bioapps/primer3_www.cgi). Cross reaction of primers with the genes was excluded by comparison of the sequence of interest with a database (Blast, US National Centre for biotechnology Information, Bethesda, MD Expression of 5 homo sapiens genes (Glyceraldehyde -3- Phosphate dehydrogenase, (GAPDH); Aldehyde dehydrogenase 3 family, member A1 (ALDH3A1); tensin 4 (TNS4); claudin 2 (CLDN2), transcript variant 1; aldo-keto reductase family 1, member B10 (aldose reductase) (AKR1B10) was determined by real time RCR as detailed above. The detection of homo sapiens GAPDH employed a pair of primers 5′-CCAGCCGAGCCACATCGCTC-3′F and 5′ -CCCCCTGCAAATGAGCCCCA-3′R prepared by Integrated DNA Technologies, expected amplicon size 370 bp. The following primer pairs were obtained from Thermo Fisher, Hanover Park, IL manufacturer, Operon Biotechnologies Inc: Homo sapiens Aldehyde dehydrogenase 3 family, member A1 (ALDH3A1) RNA was detected using a pair of primers 5′-TCAGCAGGACGAGCTCTACA-3′F and 5′-GCAGGCTCGCCATGTTCTCA-3′R; expected amplicon size 170 bp. Homo sapiens tensin 4 (TNS4) RNA was detected using a pair of primers 5′-GGACCCCAGAGGACCTTGACTCCTA-3′F and 5′-ACCACTGCGAAGGGAGCCGA-3′R;expected amplicon size 281 bp. Homo sapiens claudin 2 (CLDN2), transcript variant 1 RNA was detected using a pair of primers 5′-TGGCCTCTCTTGGCCTCCAACTTGT-3′F and 5′-GCCTGGATGTCAGCGGGCAG-3′R, amplicon size 235 bp. Homo sapiens aldo-keto reductase family 1, member B10 ( aldose reductase) (AKR1B10) RNA was detected using a pair of primers 5′-TGTGGGCCTGGGCACTTGGA-3′F and 5′-GGTCCTCCCGCTTCACAGCC-3′R, amplicon obtained by this primer pair was 173 bp. Normalized gene expression (∆∆ CT)) of (ALDH3A1), (TNS4), (CLDN2), and (AKR1B10) in E vs R cells relative to GAPDH reference gene were calculated following primer efficiency calibration for GADPH using Bio-Rad CFX Manager Software version 1.5.

### ALDH, actin, and nuclear immunofluorescent staining

ALDEFLUOR™ reagent kit was used to identify human cancer cells that express high levels of the enzyme aldehyde dehydrogenase (ALDH). Cultures were added with 5 μL of ALDEFLUOR™ reagent per milliliter of cells and incubated for 30 to 60 min at 37°C followed by storage on ice or at 4°C. An epi-fluorescent microscope and the Metamorph imaging software were used to quantify stained E and R cells. Cultures for actin and nuclear staining were fixed with 4% paraformaldehyde at 37oC for 30 min and permeabilized using 0.1% Triton × 100 for 15 min. Cultures were kept in HPLC (high performance liquid chromatography) Grade Methanol at -20°C in a closed glass vial for 15 min. DAPI was used for staining the cell nuclei. Rhodamine phalloidin (520/650, red) served as a fluorescent conjugate to bind specifically to F-actin filaments. Image-iT FX signal enhancer (Cat. No. I36933; Invitrogen) was used throughout the staining process to enhance the imaging signal-to-noise ratio and diminish non-specific antibody binding [74-76].

### Reactive oxygen species (ROS) detection and imaging

The carboxy-H2DCFDA (C25H16Cl2O9; No.: C400; Invitrogen, Molecular Probes), carboxy derivative of fluorescein, was used as cell-permeating indicator for reactive oxygen species (ROS). Mechanistically, the non-fluorescent 5-(and-6)-carboxy-2′, 7′-dichlorodihydrofluorescein diacetate (carboxy-H2DCFDA) permeates live cells to enter the cytoplasm where it is deacetylated by nonspecific intracellular esterases. In the presence of nonspecific ROS in targeted cells, the reduced fluorescein compound is oxidized and emits green fluorescence (504/530, Figure 4a) [78,79]. An optimal dye-loading concentration was empirically determined as 5.0 μM (504/530, green) for HCT-8 cell monolayers. The working solution was freshly prepared using high quality anhydrous dimethylsulfoxide (DMSO) prior to experiments, and the excess diluted probe was discarded at the end of the experiment. The phenol-red-free HCT-8 growth medium (Catalog number R8755, Sigma-Aldrich, St. Louis, MO) was removed, replaced by the working solution, and incubated at 37°C for 15 min (empirically determined as optimal). Following this incubation, the working solution was removed and replaced with pre-warmed growth medium followed by confocal microscopy imaging after 5–10 min recovery time.

### Laser confocal microscopy imaging and analysis

The actin structures and ROS expression patterns of HCT-8 cells were imaged with a Leica SP2 confocal microscopy (Leica SP2, Heidelberg, Germany) with Amira (Advanced3DVisualization and Volume Modeling) software. An inverted optical microscope (Olympus IX81, Olympus America) and a SPOT camera were used to record living cell behavior using phase-contrast microscopy. A high-resolution Piezo-controlled microscope stage was used for multi-spot time-lapse video recording. The image analysis and processing were done using ImageJ, Matlab 7, Photoshop CS2 and Microsoft Excel 2012.

### Anchorage-independent growth and viability assay

PA gels with 20 kPa stiffness were prepared on glass-slide with grids (EMS Inc.) following the procedures described above, but without any surface ECM functionalization, i.e. fibronectin or laminin (BD Bioscience). These non-functionalized surfaces prevent any specific or non-specific cell anchorage. Single E and R cells were separately cultured on gels at a starting density of 1 × 10^5^ cells per gel. Three independent repeats were carried out with 4 gels for each cell type. The anchorage-free survival of cells was confirmed by daily imaging. Care was taken during gentle medium change to minimize cell loss, though a few cells in suspension were unavoidably removed. The formation of spheroid colonies from single cells, the survival of single cells, and the growth of cells in suspension were observed and quantified with the assistance of gridded glass-slides. After 1-week of culture, a 2.0 ml aliquot of the cell suspension containing both spheroids and single cells was harvested per gel following gentle shaking to ensure unbiased sample acquisition, and examined between 2 transparent, sterile glass-slides under a light microscope. A 0.4% sterile filtered Trypan Blue solution (Sigma–Aldrich, St. Louis, MO) was applied in the cell medium to quantify the percentage of viable cells. For each 2 ml sample, 40 spots were imaged and the numbers of live/dead cells were counted. Percentage of viable cells is defined as number of viable (unstained) cells vs. total number of cells. Data for single cells and spheroids are compared respectively.

### Animal models and histological results

All animal procedures were approved by the University of Illinois Institutional Animal Care and Use Committee. Athymic nude mice (5–6 weeks old) from Charles River Laboratory were acclimated for five weeks and fed a modified AIN93A diet (calories 11%, fat 39%, carbohydrates 50%, proteins 20%). Animals were surgically injected in the spleen with either HCT-8E or HCT-8R cells. Mice were anaesthetized with isoflurane and the spleen was exteriorized via abdominal midline incision. Prior to the procedure, HCT-8E and HCT-8R cells were washed with PBS, harvested by trypsinization, and suspended in PBS. Each animal was injected with one million cells suspended in 50 μl of PBS. The muscle wall of the abdomen was closed with absorbable suture. The skin incision was then closed using a sterile stainless steel wound clip. Animals were sacrificed 9–10 weeks after cancer cell injection. Mice were euthanized under anesthesia by exsanguination. During the necropsy, the liver, lungs, spleen and other implantation sites were sampled, fixed in 10% buffered formalin and embedded in paraffin. Tissue sections (5 μm) were stained with H&E. Six representative sections of the liver (left lateral lobe, right and left median lobes) and one section of the spleen, lungs, and all other tumor implantation sites were evaluated histologically by a certified veterinary pathologist.

## Abbreviations

E-cell: Adhesive epithelial type; R-cell: Rounded dissociated type; RNA-seq: RNA Sequencing; ROS: Reactive oxygen species; PS: Polystyrene; PA: Polyacrylamide; ALDH3A1: Aldehyde dehydrogenase 3 family member A1; TENS4: Tensin 4; CLDN2: Claudin 2; AKR1B10: Aldo-keto reductase family 1, member B10; ALDH: Aldehyde dehydrogenase; AFM: Atomic force microscopy; GADPH: Glyceraldehyde -3- Phosphate dehydrogenase; EMT: Epithelial-to-mesenchymal transition; FPKM: Fragments Per Kilobase of exon per Million fragments mapped; DIC: Differential interference contrast.

## Competing interests

All authors declare that they have no competing interests.

## Authors’ contributions

XT participated in the design of the study, carried out most of the *in vitro* experiments, AFM, statistical analysis and drafted the manuscript. TBK carried out the cell culture and qPCR validation experiments and helped draft the manuscript. QL carried out the animal trial, data analysis and helped to draft the manuscript. SA performed all technical aspects of the RNA-seq experiments and assisted in the bioinformatics analyses in collaboration with the University of Illinois Roy J. Carver Biotechnology Center. SL histologically evaluated the paraffin-embedded slides and helped with the editing of the manuscript. HC designed and participated in the animal study and helped to draft the manuscript. MA and WWL assisted in initial study design of the RNA-seq and qPCR validation experiments. TAF and MSK made substantial contribution to the conception of the study, participated in its design and coordination, interpretation of the RNA-seq data and helped to draft the manuscript. All authors read and approved the final manuscript.
